# Chronic social defeat stress increases the amounts of 12-lipoxygenase lipid metabolites in the nucleus accumbens of stress-resilient mice

**DOI:** 10.1038/s41598-022-15461-7

**Published:** 2022-07-05

**Authors:** Satoshi Akiyama, Hirotaka Nagai, Shota Oike, Io Horikawa, Masakazu Shinohara, Yabin Lu, Takashi Futamura, Ryota Shinohara, Shiho Kitaoka, Tomoyuki Furuyashiki

**Affiliations:** 1 Division of Pharmacology, Graduate School of Medicine, Kobe University, 7-5-1 Kusunoki-cho, Chuo-ku, Kobe, 650-0017 Japan; 2 Department of CNS Research, Otsuka Pharmaceutical Co., Ltd., Tokushima, 771-0192 Japan; 3 Japan Agency for Medical Research and Development, Tokyo, 100-0004 Japan; 4 Department of Community Medicine and Social Healthcare Science, Division of Epidemiology, Graduate School of Medicine, Kobe University, Kobe, 650-0017 Japan; 5 The Integrated Center for Mass Spectrometry, Graduate School of Medicine, Kobe University, Kobe, 650-0017 Japan; 6 Present Address: Department of Pharmacology, School of Medicine, Hyogo Medical University, Nishinomiya, 663-8501 Japan

**Keywords:** Neuroscience, Emotion, Molecular neuroscience, Social behaviour, Stress and resilience

## Abstract

Severe and prolonged social stress induces mood and cognitive dysfunctions and precipitates major depression. Neuroinflammation has been associated with chronic stress and depression. Rodent studies showed crucial roles of a few inflammation-related lipid mediators for chronic stress-induced depressive-like behaviors. Despite an increasing number of lipid mediators identified, systematic analyses of synthetic pathways of lipid mediators in chronic stress models have not been performed. Using LC–MS/MS, here we examined the effects of chronic social defeat stress on multiple synthetic pathways of lipid mediators in brain regions associated with stress susceptibility in mice. Chronic social defeat stress increased the amounts of 12-lipoxygenase (LOX) metabolites, 12-HETE and 12-HEPE, specifically in the nucleus accumbens 1 week, but not immediately, after the last stress exposure. The increase was larger in stress-resilient mice than stress-susceptible mice. The S isomer of 12-HETE was selectively increased in amount, indicating the role of 12S-LOX activity. Among the enzymes known to have 12S-LOX activity, only Alox12 mRNA was reliably detected in the brain and enriched in brain endothelial cells. These findings suggest that chronic social stress induces a late increase in the amounts of 12S-LOX metabolites derived from the brain vasculature in the nucleus accumbens in a manner associated with stress resilience.

## Introduction

Severe and prolonged social stress induces mood and cognitive dysfunctions and predisposes to mental illnesses such as major depression^1^. Biological mechanisms of chronic stress have been studied using its rodent models such as repeated social defeat stress and chronic unpredictable stress^2–5^. Rodent studies identified chronic stress-induced structural and functional changes of neurons in brain areas related to emotion and analyzed the molecular mechanisms involving neurotransmitters^5–11^. However, recent studies indicated crucial roles of non-neuronal brain cells in chronic stress and depression. Multiple types of chronic stress reportedly activate microglia that secrete inflammation-related mediators for neuronal and behavioral changes^5,12,13^. Chronic social defeat stress also impairs the blood–brain barrier that exacerbates neuroinflammation via the leakage of blood-borne cytokines^14,15^. In addition, clinical studies suggested elevated inflammatory states in the brain and blood of depressive patients^16,17^.

Despite the emerging evidence of the interaction between neurons and non-neuronal cells in chronic stress and depression, its molecular mechanisms remain poorly understood. Lipid mediators, including prostaglandins (PGs) and leukotrienes (LTs), play fundamental roles in inflammation^18,19^. Lipid mediators are synthesized by sequential actions of synthases from poly-unsaturated fatty acids (PUFAs), including arachidonic acid (AA), an ω6 PUFA, and eicosapentaenoic acid (EPA) and docosahexaenoic acid (DHA), ω3 PUFAs, after cleaved from phospholipids^20^. Previous rodent studies showed roles of PGE_2_ and cysteinyl LTs, both of which are derived from AA, and their receptors in chronic stress-induced depressive-like behaviors^5,19,21^. These mediators are thought to mediate neuron-glia interaction for the behavioral changes^22^. Clinical studies also showed that celecoxib that inhibits PG synthesis augments therapeutic effects of antidepressants in depressive patients^23^.

Recent technical advances in liquid chromatography-tandem mass spectrometry (LC–MS/MS) have led to identifying many new lipid metabolites in biological samples^24,25^. Their functional analyses have forged the concept of pro-resolving lipid mediators that are synthesized later than proinflammatory lipid mediators to resolve inflammatory responses^26^. Most pro-resolving lipid mediators are synthesized from EPA and DHA by lipoxygenases (LOXs). Diets rich in ω3 PUFAs, EPA and DHA, have been reported to prevent chronic stress-induced depressive-like behaviors in mice and may also mitigate symptoms in depressive patients^27–29^. Such accumulating evidence implies the involvement of various lipid mediators derived from EPA and DHA along with AA-derived ones in chronic stress and depression. However, synthetic pathways of these multiple lipid mediators have not systematically been analyzed in rodent models of chronic stress.

In this study, we examined the effects of chronic social defeat stress on multiple synthetic pathways of lipid mediators in brain regions associated with stress susceptibility in mice. Using LC–MS/MS, we performed a comprehensive analysis of pathway markers of lipid mediator synthesis from AA, EPA, and DHA and found that chronic social defeat stress increased the amounts of 12-LOX metabolites in the nucleus accumbens. Their increase was higher in stress-resilient mice that did not develop depressive-like behavior than stress-susceptible mice. Thus, this study identified brain lipid mediators associated with stress resilience.

## Methods

### Animals

Adult male C57BL/6N mice of 7–10 weeks old and male ICR mice retired from breeding were purchased from Japan SLC (Shizuoka, Japan). Mice were maintained in the animal facility with constant temperature and humidity on a 12 h light/12 h dark cycle with food and water available ad libitum. Mice were first housed in groups of 4–5 mice per cage and then singly housed before behavioral experiments. All procedures for animal care and use were in accordance with the ARRIVE guidelines and the National Institutes of Health Guide for the Care and Use of Laboratory Animals and were approved by the Animal Care and Use Committees of Kobe University Graduate School of Medicine.

### Chronic social defeat stress

**Figure 1:**
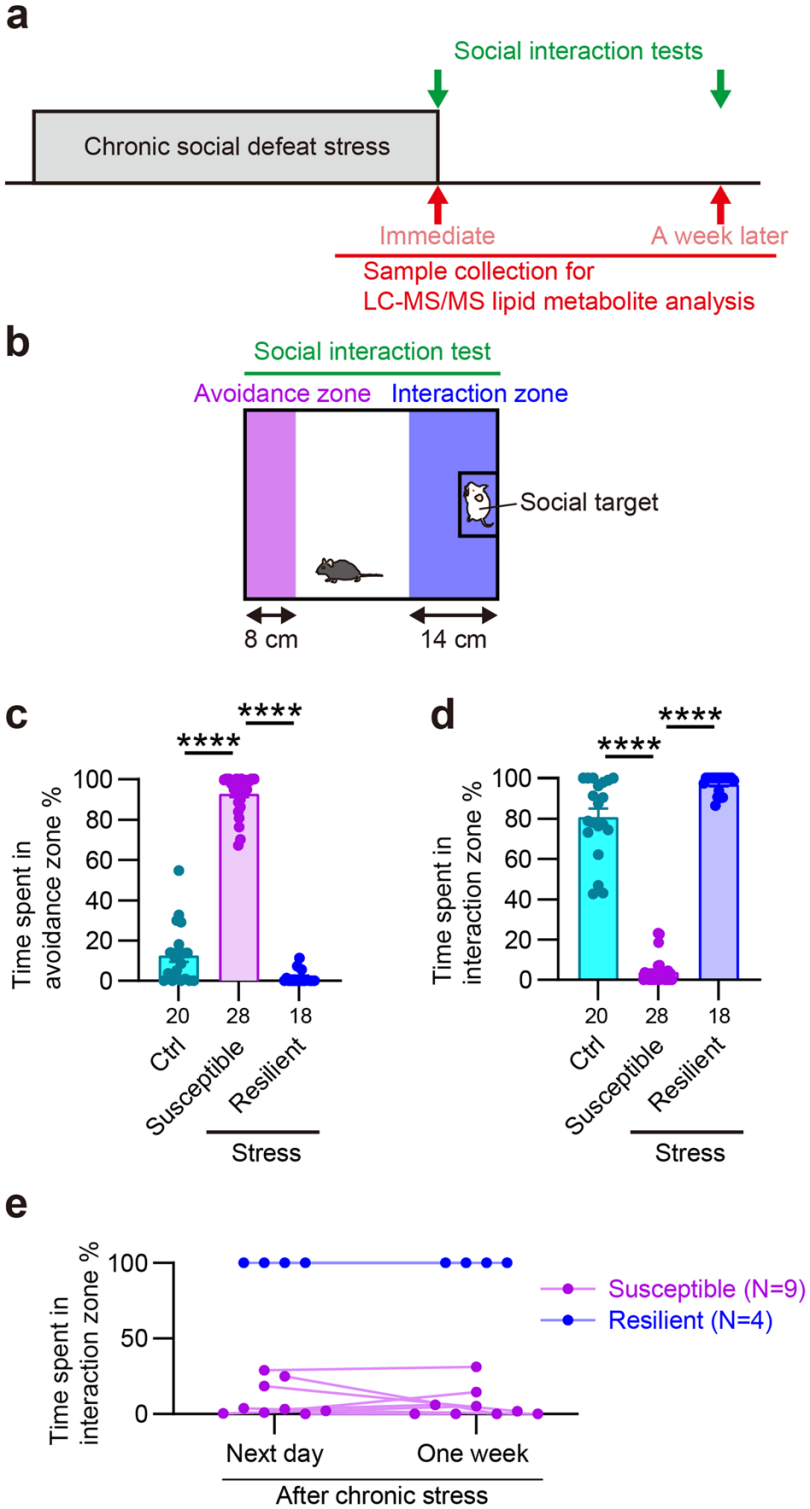
Chronic social defeat stress induced depressive-like behavior in stress-susceptible mice and not in stress-resilient mice. (**a**) An experimental schedule. Mice received chronic social defeat stress composed of repeated exposure to social defeat stress for 10 min daily for 10 consecutive days. Control and stressed mice received the social interaction test to examine chronic social defeat stress-induced social avoidance, an index of depressive-like behavior. Multiple brain regions and blood plasma were collected for lipid metabolite analysis immediately or 1 week after the last stress exposure. (**b**) A behavioral chamber used for the social interaction test. An area close to a social target enclosed in a metal meshwork is defined as the interaction zone and the opposite area as the avoidance zone. (**c**,**d**) The time spent in the avoidance zone (**c**) or the interaction zone (**d**) during the social interaction test. (**e**) The behavioral results of the social interaction test on the next day and 1 week after chronic stress. The number of samples is shown below each bar or indicated next to the legend. See “Methods” for definition of the mice. Values are expressed as means ± SEM. ****P < 0.0001 for Tukey’s multiple comparisons test following one-way ANOVA.

Chronic social defeat stress was performed as previously described with minor modifications^4,9,12^ (Fig. 1a). We first selected male ICR mice based on their aggressiveness to a novel male C57BL/6N mouse for 3 min daily for 3 days. The aggressiveness was assessed by the latency and frequency of attacks during the observation period, and only the ICR mice with stable aggression were used as aggressor mice. Male C57BL/6N mice were divided into two groups. A mouse in the chronic stress group was transferred to the home cage of a male aggressor ICR mouse and received aggression for 10 min daily for 10 consecutive days. After each 10 min bout of social defeat, stressed mice were returned to their home cages and left undisturbed until the next stress exposure or the social interaction test. A mouse in the control group was transferred to a novel empty cage without an aggressor ICR mouse.

### Social interaction test

The social interaction test was performed as previously described^4,9,12,30^. One day before the first stress exposure, an experimental mouse was habituated for 150 s to an open rectangle chamber (30 cm × 40 cm) with an empty metal meshwork placed at one end. In the social interaction test, the mouse was returned to the same chamber with a novel ICR mouse enclosed in the metal meshwork and allowed to explore for 150 s. The locomotion of the mouse was video-recorded and analyzed post hoc using the SMART video tracking software (PanLab Harvard Apparatus, Holliston, MA, USA). The areas at one side of the ICR mouse and the opposite side were defined as the interaction zone and the avoidance zone, respectively (Fig. 1b). The time spent in each zone was determined to evaluate chronic social defeat stress-induced social avoidance. Mice which spent more than half of the time in the social avoidance zone were defined as stress-susceptible mice and the others as stress-resilient mice. In the experiments to examine the effect of chronic social defeat stress on lipid metabolites 1 week later, stressed mice received the social interaction test on the next day after the last (i.e., tenth) stress exposure. The mice were then left undisturbed until 6–7 days later when they were sacrificed for collecting samples. To examine the sustained effects of stress on behaviors, some mice that were not used for lipid metabolite analysis received the social interaction test on the next day and 1 week after the last stress exposure. In the experiments to examine the immediate effect of the stress, we performed the social interaction test on the next day after the eighth stress exposure and collected samples immediately after the tenth stress exposure. The mice used for lipid metabolite analyses in this study include those used for our previous behavioral analyses^4^.

### Lipid metabolite analysis

For lipid metabolite analyses, mice were deeply anesthetized with intraperitoneal injection of sodium pentobarbital. We collected their blood through the heart using a syringe filled with 10 μL of 300 mM EDTA and removed their brains after decapitation. The blood samples were centrifuged at 3000 rpm for 10 min at 4 °C. The supernatant was recovered as blood plasma. The brains were surgically dissected based on Allen Brain Atlas, and brain tissues of the prefrontal cortex, the nucleus accumbens, the dorsal striatum, the amygdala, and the hippocampus were obtained. The obtained samples were kept frozen at − 80 °C until use. Brain tissue and blood plasma samples were added with an excess volume of ice-cold methanol and deuterated internal standards such as d4-LTB_4_, d8-5-HETE, d4-PGE_2_, and d5-RvD_2_ (500 pg each). Brain tissue samples were further homogenized using zirconia beads. The samples were incubated for 1 h at 4 °C and centrifuged at 15,000 rpm for 5 min at 4 °C. Recovered supernatants were extracted by solid phase extraction on C18 columns, and extracted lipid metabolites were measured by LC–MS/MS. LC–MS/MS analyses were performed, as previously described^24^. For chiral analysis, a Chiralpak AD-RH column was used with isocratic acetonitrile/water/formic acid 65:35:0.1 (v/v/v) at 0.2 mL/min flow rate, as previously described^31^. To increase the signal-to-noise ratio, we pooled the tissues of the same brain region, except the prefrontal cortex, obtained from three mice within the same group (i.e., control mice, stress-susceptible mice, and stress-resilient mice). Brain tissues of the prefrontal cortex and blood plasma samples were individually analyzed. Some measurements were excluded when their peaks in mass spectrum were not well resolved. The amount of each metabolite was normalized to its average in control mice in each experimental cohort for further analyses.

### In situ hybridization

Eight-week-old C57BL/6N mice were transcardially perfused with phosphate buffer containing 4% paraformaldehyde following a flush of ice-cold D-PBS. Their brains were harvested, post-fixed in the same fixative, and cryoprotected in 30% sucrose in D-PBS at 4 °C overnight. The brains were frozen in OCT compound (Sakura Finetek) and then coronally cut at 15-μm thickness by cryostat (Leica). The brain sections were processed for RNAscope (Advanced Cell Diagnostics, Newark, CA) according to the manufacturer’s instruction. The brain sections were treated with probes for mouse Alox12 (RNAscope Probe-Mm-Alox12, Cat No. 539771) or with negative control probes (RNAscope Negative Control Probe-DapB, Cat No. 310043). Signals were visualized using diaminobenzidine. The brain sections were counterstained with cresyl violet, dehydrated, and coverslipped. Bright-field images of the brain sections were obtained using the BZ-X710 microscope (Keyence, Osaka, Japan).

### Quantitative RT-PCR

Nucleus accumbens tissues were collected as described above in the “Lipid metabolite analysis” section. The tissues were kept frozen until use. RNA was extracted from these tissues using a column-based RNA purification method (NucleoSpin RNA XS, Macherey-Nagel), and cDNA was then synthesized using PrimeScript RT reagent kit with gDNA eraser (RR047A, Takara). Quantitative PCR was performed using PowerTrack SYBR Green Master Mix (A46012, Thermo Fischer) on the CFX384 system (Bio-Rad). The sequences of the primers used are as follows: β-actin forward: 5′-CAT TGC TGA CAG GAT GCA GAA GG-3′, β-actin reverse: 5′-TGC TGG AAG GTG GAC AGT GAG G-3′, Alox5 forward: 5′-TCT TCC TGG CAC GAC TTT GCT G-3′, Alox5 reverse: 5′-GCA GCC ATT CAG GAA CTG GTA G-3′, Alox12 forward: 5′-TGG ACT TTG AAT GGA CGT TG-3′, Alox12 reverse: 5′-TTC TCG GCC AAG GCA CTC T-3′, Alox12e forward: 5′-GTC ACT GAG GTT GGA CTG CTT G-3′, Alox12e reverse: 5′-GTG TAG ATG CGT GCT GAC CAG-3′, Alox15 forward: 5′-CTG TTA CCG ATG GGT TCA GG-3′, Alox15 reverse: 5′-AGT TCC TCC TCC CTG TGG TT-3′.

### Forced swim test

Eight-week-old male C57BL/6N mice were intraperitoneally injected with imipramine (2 or 20 mg/kg) or with saline and subjected to the forced swim test 30 min later. The forced swim test was performed as previously described^32^. Briefly, a mouse was placed into a cylinder with enough water so that the mouse could not touch the bottom with its paws. Water temperature was adjusted to 25 °C. Behaviors were video-recorded and analyzed post hoc using the SMART video tracking software. The duration of floating behavior without struggling was determined as immobility time, a behavioral index of depressive-like behavior^32^. The mice were sacrificed 30 min after the forced swim test for lipid metabolite analysis. Naïve mice without either intraperitoneal injection or the forced swim test were also analyzed.

### Statistical analyses

Data are shown as means ± SEM. Statistical analyses were performed by Prism 9.3 software (GraphPad Software, San Diego, CA, USA). *P* values less than 0.05 were considered significant. For lipid metabolite analyses, two-way ANOVA followed by Tukey’s multiple comparisons test was used to compare values with two independent factors (Figs. 3a–e, 4a,b, 7c and Supplementary Fig. 2b). Pearson’s correlation test was used to evaluate correlation between two independent values (Figs. 5a–j and 6a–e). Since a few strong outliers erroneously generated statistical significance in these analyses, we excluded the outliers detected using the ROUT method at the 1% false discovery rate. It should be noted that there were no such outliers in the measurements of 12-HETE and 12-HEPE in the nucleus accumbens used to derive the conclusions of this study. For the remaining data, one-way ANOVA followed by Tukey’s multiple comparisons test was used to compare more than two groups (Figs. 1c,d and 8a, Supplementary Fig. 2a).

## Results

### Chronic stress induces a late increase in the amounts of brain lipid metabolites associated with resilience

**Figure 2:**
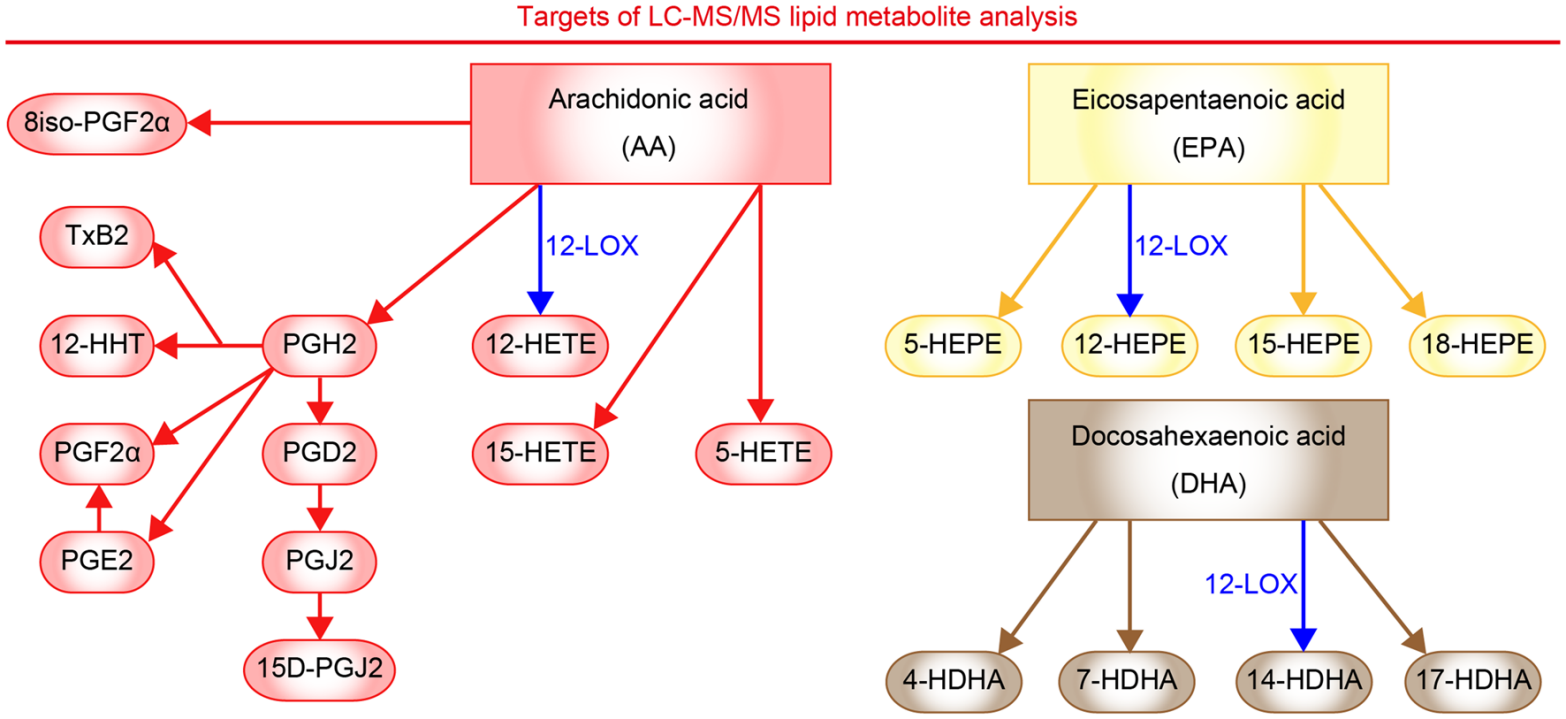
Diagram of synthetic pathways of lipid metabolites examined in the present study. Metabolic reactions mediated by 12-lipoxygenase (LOX) is highlighted. See the text for abbreviations of lipid metabolites.

**Figure 3:**
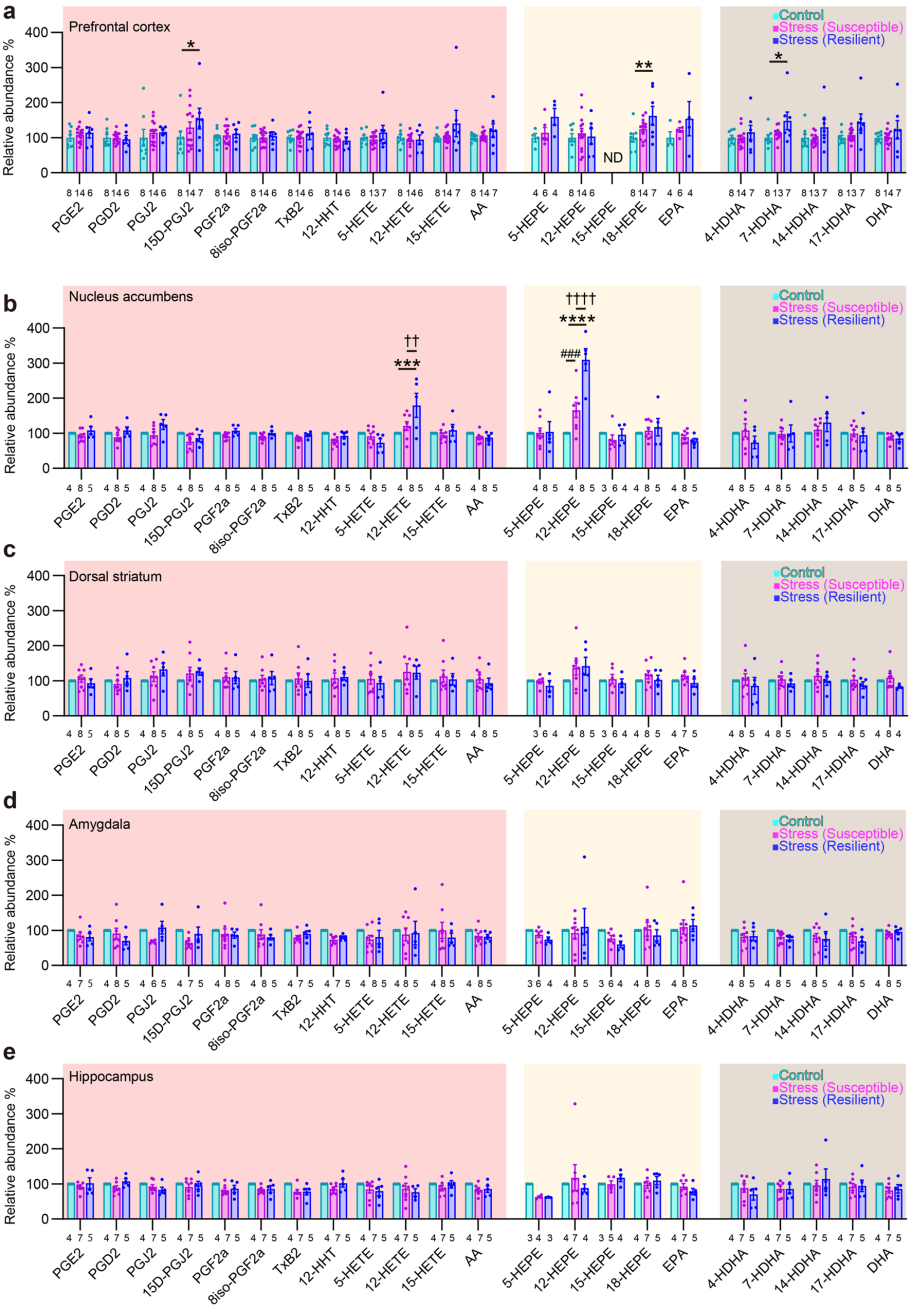
Chronic social defeat stress increased the amounts of brain lipid metabolites in stress-resilient mice. (**a**–**e**) The amounts of each metabolite in the prefrontal cortex (**a**), the nucleus accumbens (**b**), the dorsal striatum (**c**), the amygdala (**d**), and the hippocampus (**e**) in control mice, stress-susceptible mice, and stress-resilient mice. The lipid metabolites related to AA, EPA and DHA are shown on pink, yellow and beige backgrounds, respectively. The values were normalized to those in control mice. 15-HEPE in the prefrontal cortex was not detected (ND). The number of samples is shown below each bar. Values are expressed as means ± SEM. Tukey’s multiple comparisons test was used for statistical analyses. ^###^*P* < 0.001 for the comparison between control and stress-susceptible mice, *P < 0.05, **P < 0.01, ***P < 0.001, ****P < 0.0001 for the comparison between control and stress-resilient mice, and ^††^*P* < 0.01, ^††††^*P* < 0.0001 for the comparison between stress-susceptible mice and stress-resilient mice. See the text for abbreviations of lipid metabolites.

**Figure 4:**
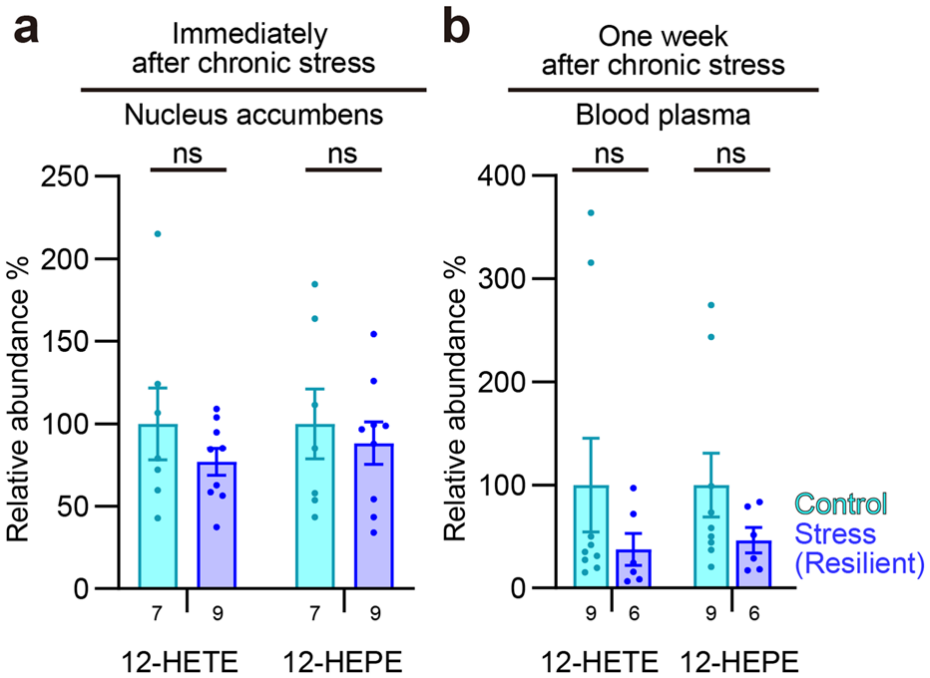
Stress-resilient mice did not show changes in 12-LOX metabolites in the nucleus accumbens immediately after chronic social defeat stress or in the blood plasma 1 week later. The amounts of 12-HETE and 12-HEPE in control mice and stress-resilient mice are shown after normalized to respective control values. The number of samples is shown below each bar. *ns* not significant for Tukey’s multiple comparisons test.

We sought synthetic pathways of brain lipid mediators associated with individual variability of stress susceptibility. We subjected adult male C57BL/6N mice to chronic social defeat stress for 10 days and evaluated their social behaviors to a caged, novel male ICR mouse in the social interaction test on the next day (Fig. 1a,b). Some stressed mice showed social avoidance, mostly staying in the avoidance zone, whereas others spent most time interacting with the social target (Fig. 1c,d), as previously reported^4,33^. We named these groups of mice stress-susceptible and stress-resilient mice, respectively. The social behaviors of these mice remained the same over 1 week after the stress (Fig. 1e). To avoid the direct effects of stress exposure, we first examined lipid metabolite profiles in the brain 1 week after the last stress exposure. We chose several brain regions reportedly involved in stress-induced behavioral changes for systematic analyses of pathway markers of lipid mediator synthesis from AA, EPA, and DHA using LC–MS/MS (Fig. 2). Detected AA metabolites were prostaglandin (PG) E_2_, PGD_2_, PGJ_2_, 15D-PGJ_2_, PGF_2α_, 8iso-PGF_2α_, thromboxane B_2_ (TxB_2_), 12-hydroxyheptadecatrienoic acid (12-HHT), 5-hydroxyeicosatetraenoic acid (5-HETE), 12-HETE, and 15-HETE. Detected EPA metabolites were 5-hydroxyeicosapentaenoic acid (5-HEPE), 12-HEPE, 15-HEPE, and 18-HEPE. Detected DHA metabolites were 4-hydroxy-docosahexaenoic acid (4-HDHA), 7-HDHA, 14-HDHA, and 17-HDHA. Chronic social defeat stress increased the amounts of a subset of these lipid metabolites in specific brain regions such as 15D-PGJ_2_, 18-HEPE, and 7-HDHA in the prefrontal cortex and 12-HETE and 12-HEPE in the nucleus accumbens (Fig. 3a–e). Notably, 12-HETE and 12-HEPE increased in stress-resilient mice significantly more than stress-susceptible mice. No alteration was found in the dorsal striatum, the amygdala, or the hippocampus. Notably, we did not find the immediate effects of chronic social defeat stress on the lipid metabolites that would increase 1 week later (Fig. 4a), suggesting their late increase after the stress. Although brain lipid metabolites might be derived from the blood, 12-HETE and 12-HEPE in blood plasma were unaltered 1 week after the stress (Fig. 4b). These findings suggest that chronic social defeat stress induced a late increase in the synthesis of lipid metabolites associated with resilience in the brain.

### Nucleus accumbens has distinct regulation of lipid mediator synthesis after stress

**Figure 5:**
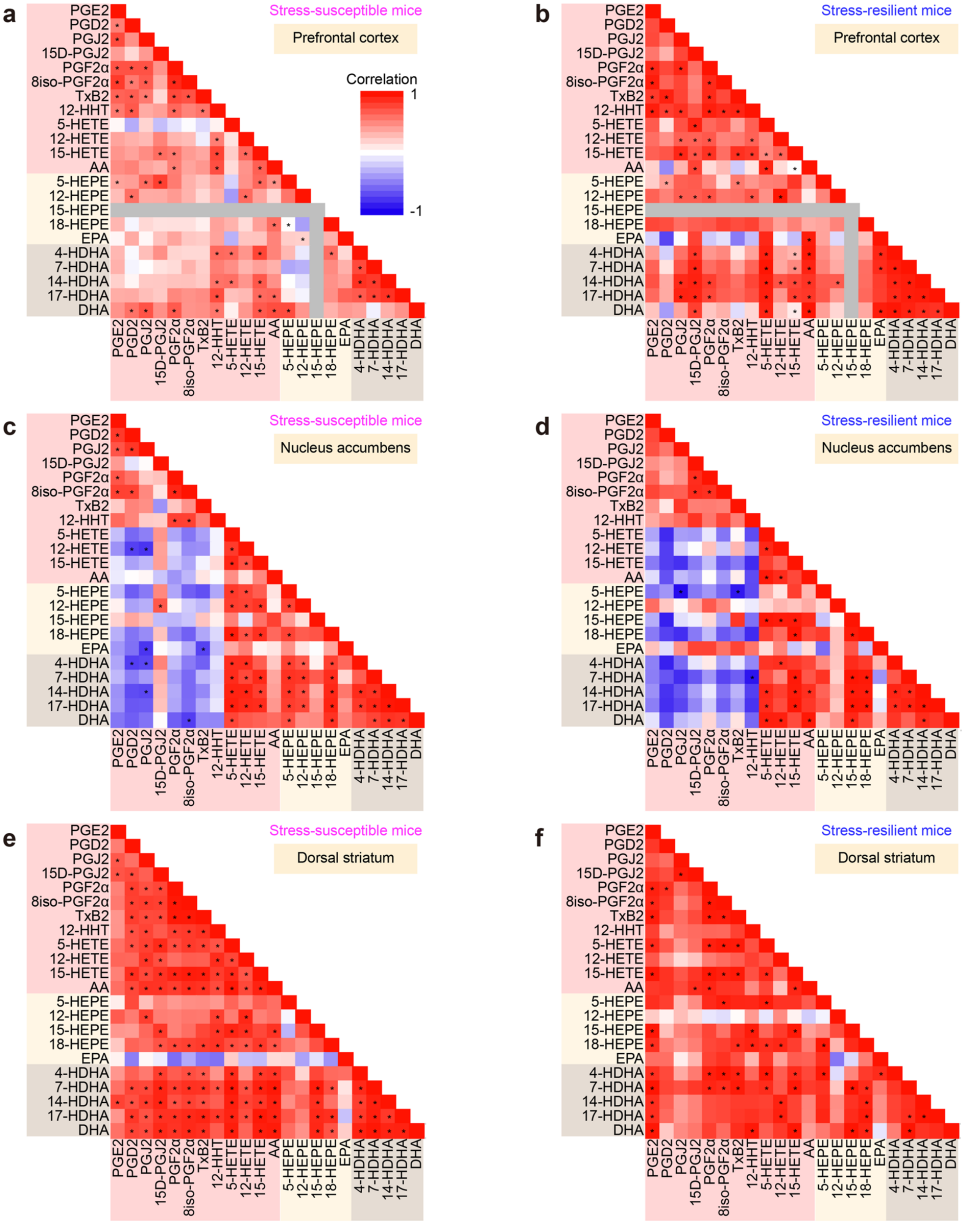

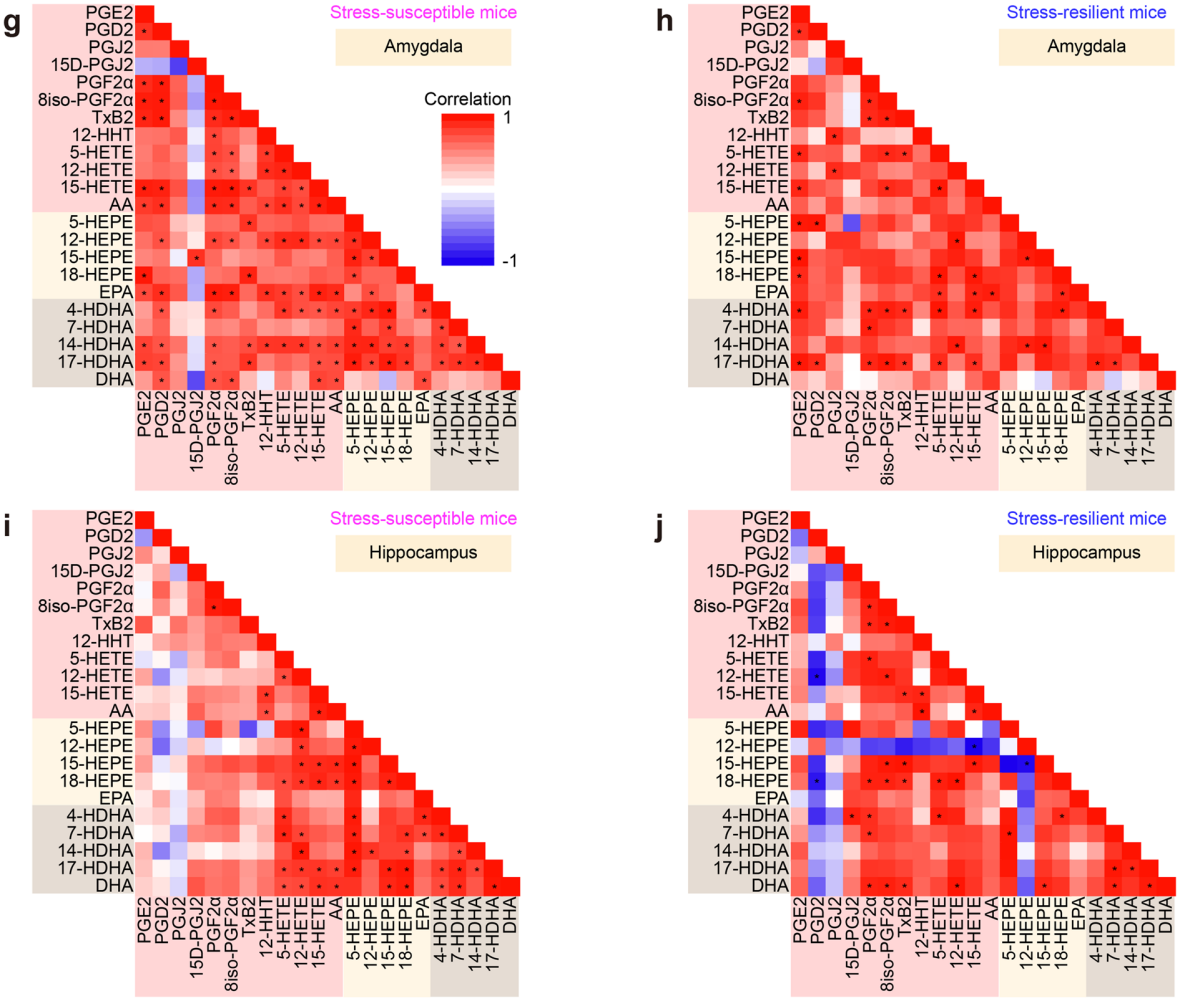
The nucleus accumbens showed a distinct pattern of the correlations between lipid metabolites. Color-coded correlation matrices of the amounts of lipid metabolites in the prefrontal cortex (**a**,**b**), the nucleus accumbens (**c**,**d**), the dorsal striatum (**e**,**f**), the amygdala (**g**,**h**), and the hippocampus (**i**,**j**) of stress-susceptible mice (**a**,**c**,**e**,**g**,**i**) and stress-resilient mice (**b**,**d**,**f**,**h**,**j**) are shown. Note that only the nucleus accumbens shows negative correlations in the pairs between a COX metabolite and a LOX metabolite in both stress-susceptible mice and stress-resilient mice. The same color coding is applied to all matrices. Color bars are shown in (**a**) and (**g**). *P < 0.05 for significant correlation by Pearson’s correlation test.

Synthetic pathways of multiple lipid metabolites may be coupled either positively or negatively by sharing and competing synthetic enzymes and precursors. We analyzed correlations between the amounts of lipid metabolites in respective brain regions of stressed mice. The nucleus accumbens showed a distinct pattern from the other brain regions. In the prefrontal cortex, the dorsal striatum, the amygdala, and the hippocampus, most pairs of lipid metabolites showed positive correlations, regardless of whether they share synthetic enzymes or precursors (Fig. 5a,b, e–j). By contrast, in the nucleus accumbens, the pairs between a cyclooxygenase (COX) metabolite (e.g., PGE_2_, PGD_2_, PGJ_2_, PGF_2α_, 8iso-PGF_2α_, and TxB_2_) and a lipoxygenase (LOX) metabolite (e.g., 5-HETE, 12-HETE, 15-HETE, 5-HEPE, 18-HEPE, 4-HDHA, 7-HDHA, 14-HDHA, and 17-HDHA) appeared to be negatively correlated, whereas the pairs within COX or LOX metabolites still showed positive correlations (Fig. 5c,d). This nucleus accumbens-specific pattern was observed in both stress-susceptible and stress-resilient mice. These findings suggest that the nucleus accumbens has distinct regulation of lipid mediator synthesis in the stressed brain.

### 12S-HETE amounts increase in the nucleus accumbens of stress-resilient mice

**Figure 6:**
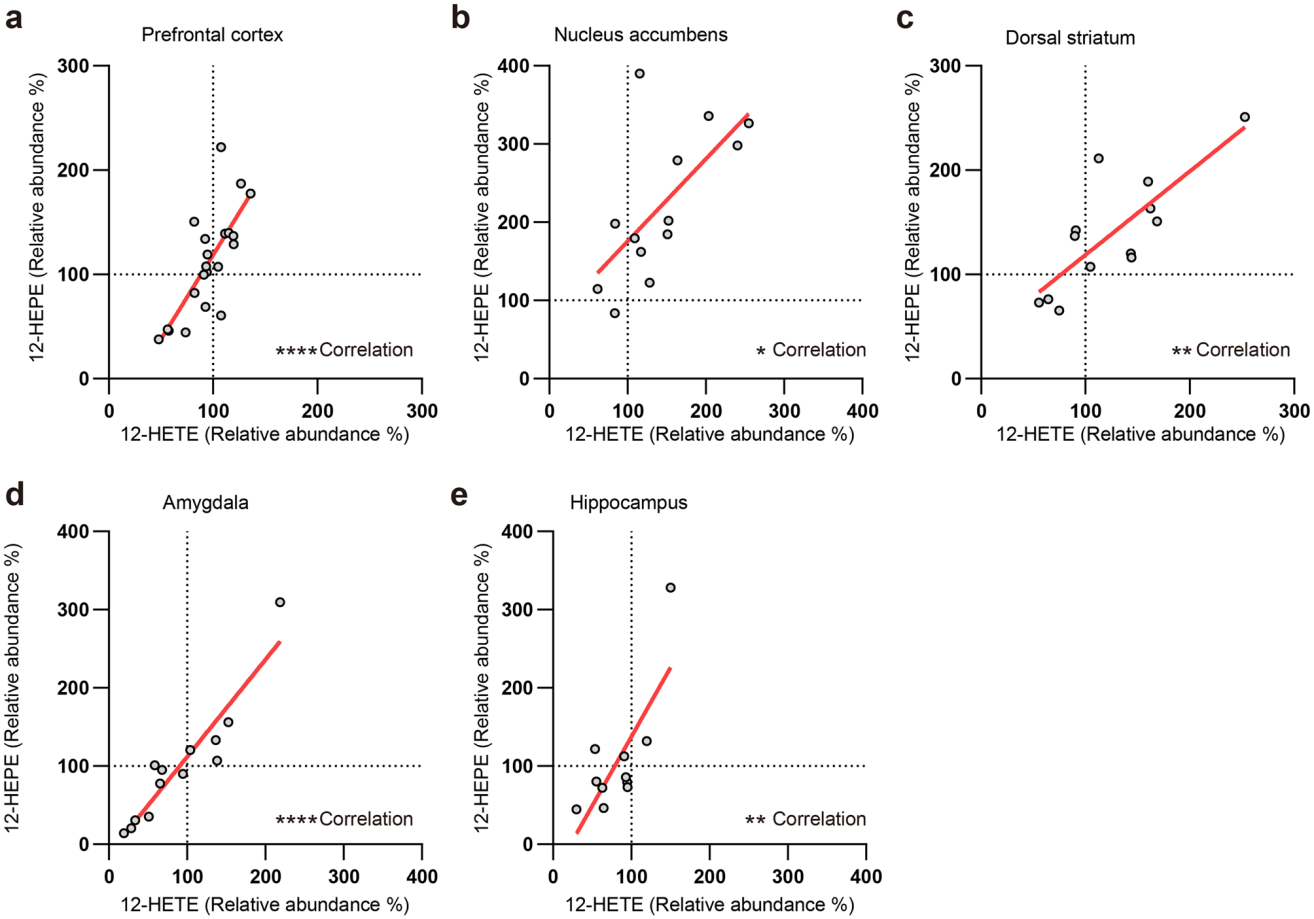
The amounts of 12-HETE and 12-HEPE were positively correlated in multiple brain regions after chronic social defeat stress. The relationship between the amounts of 12-HETE and 12-HEPE in the prefrontal cortex (**a**), the nucleus accumbens (**b**), the dorsal striatum (**c**), the amygdala (**d**), and the hippocampus (**e**) after chronic social defeat stress are shown. The values of stress-susceptible mice and stress-resilient mice were combined for this analysis. Circles represent the values of individual samples, and red lines indicate regression lines. *P < 0.05, **P < 0.01, ****P < 0.0001 for significant correlation by Pearson’s correlation test.

**Figure 7:**
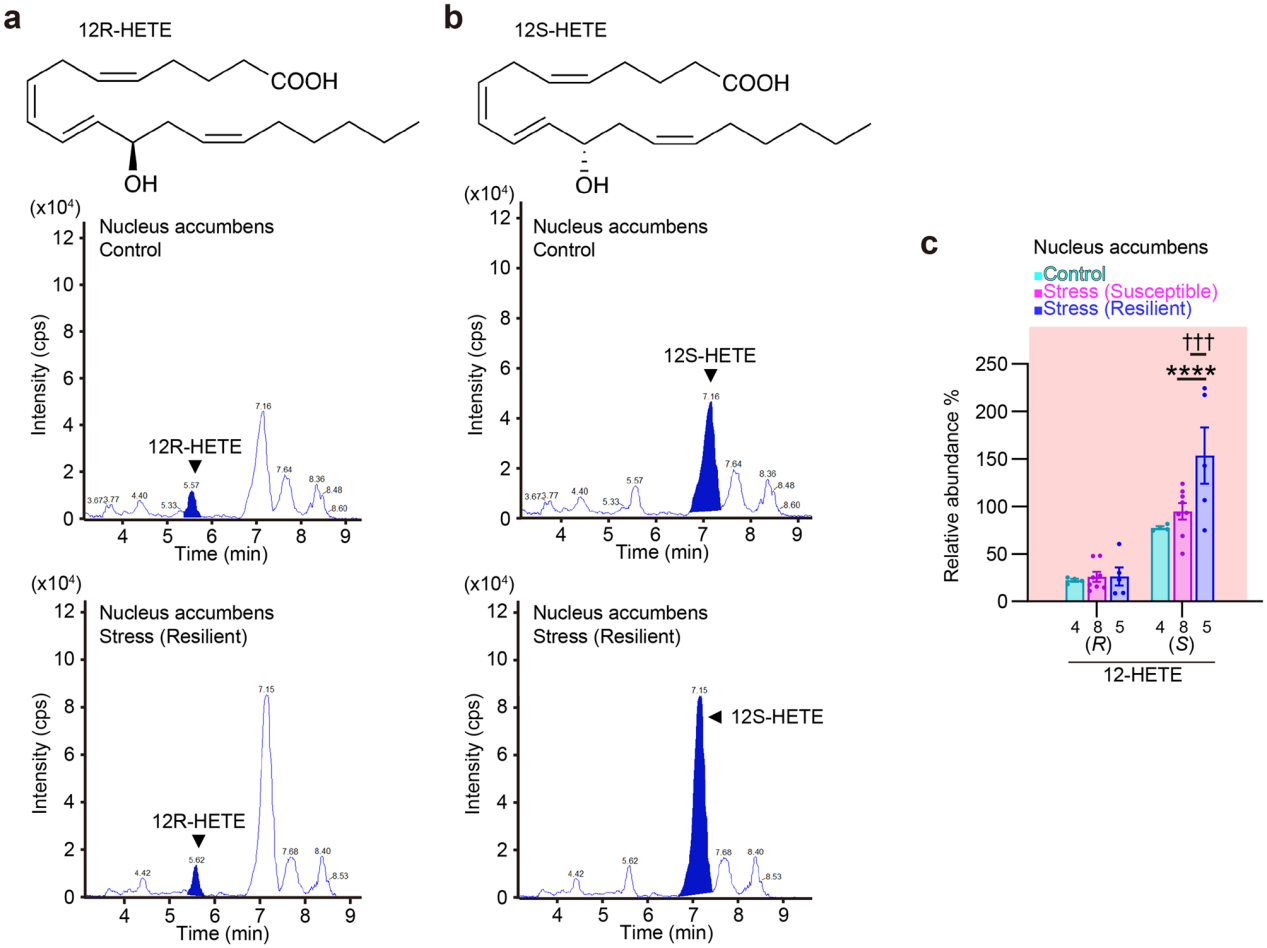
Chronic social defeat stress increased the amount of the S isomer of 12-HETE in the nucleus accumbens. (**a**,**b**) Representative mass spectra of the R and S isomers of 12-HETE (**a** and **b**, respectively) in the nucleus accumbens of control and stress-resilient mice. The peaks of 12R-HETE and 12S-HETE are filled in blue. (**c**) The amounts of the R and S isomers of 12-HETE in the nucleus accumbens of control, stress-susceptible, and stress-resilient mice. The number of samples is shown below each bar. Values are expressed as means ± SEM. Tukey’s multiple comparisons test was used for statistical analyses. ****P < 0.0001 for the comparison between control and stress-resilient mice and ^†††^*P* < 0.001 for the comparison between stress-susceptible and stress-resilient mice.

We further analyzed 12-HETE and 12-HEPE, both of which were increased in amount in the nucleus accumbens after chronic social defeat stress and are known to be synthesized by the enzymatic activity of 12-lipoxygenase (12-LOX)^34^. The amounts of 12-HETE and 12-HEPE were positively correlated in all the brain regions analyzed, including the nucleus accumbens, in stressed mice (Fig. 6a–e). Nonetheless, 12-HETE and 12-HEPE are chiral molecules composed of R and S isomers. Their R and S isomers are synthesized by different enzymes and have different biological functions^35^. Thus, we determined the amounts of each isomer in the nucleus accumbens. Our chiral analysis reliably detected R and S isomers of 12-HETE (Fig. 7a,b), but less reliably 12-HEPE isomers (data not shown), whose amounts were lower than those of 12-HETE (1.70 ± 0.85, 1.67 ± 0.59, and 2.91 ± 1.30 pg/mg tissue for 12-HETE and 0.020 ± 0.010, 0.030 ± 0.011, and 0.078 ± 0.035 pg/mg tissue for 12-HEPE in control mice, stress-susceptible mice, and stress-resilient mice, respectively). We found that the S isomer was dominant in 12-HETE and that its amounts selectively increased in stress-resilient mice (Fig. 7c). Thus, the amount of the S isomer 12S-HETE was increased in the nucleus accumbens of stress-resilient mice. The chiral specificity of 12-HETE suggests the association of 12S-LOX activity in the nucleus accumbens with stress resilience.

**Figure 8:**
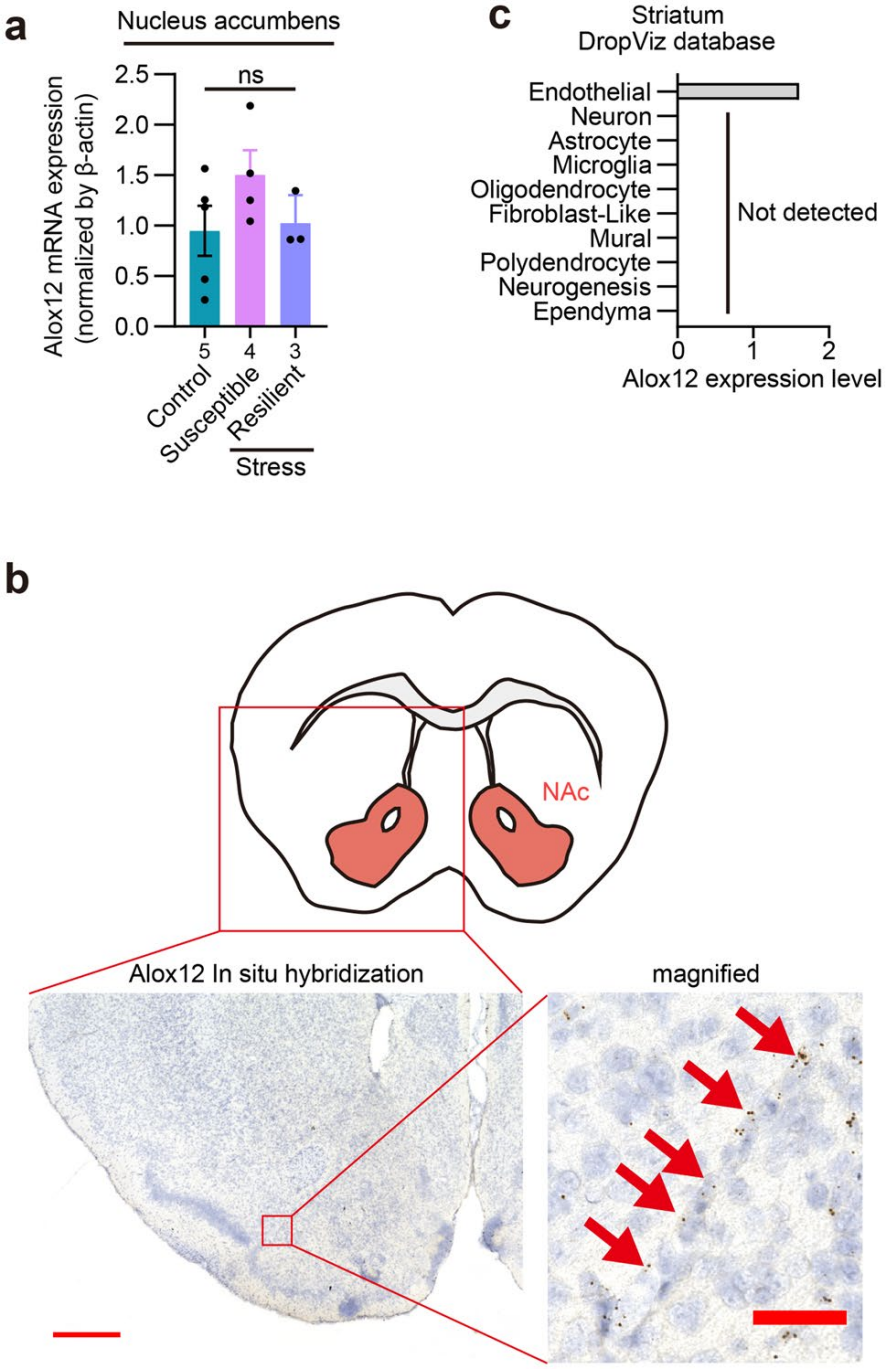
Alox12 was expressed in blood vessel-like structures in the brain. (**a**) mRNA levels of Alox12 in the nucleus accumbens of control, stress-susceptible, stress-resilient mice. The values were determined by qRT-PCR and normalized by β-actin mRNA. The number of samples is shown below each bar. ns, not significant for Tukey’s multiple comparisons test. (**b**) Representative signals of in situ hybridization for Alox12 mRNA in the nucleus accumbens. Counterstaining was performed by Nissl staining. Brown dots represent signals. Signals on a blood vessel-like structure are indicated by red arrows. Scale bars: 200 μm and 50 μm for left and right images, respectively. (**c**) Alox12 mRNA expression in endothelial cells in the striatum. Publicly available data of single cell RNA-seq were analyzed with the interactive online software DropViz.

Multiple enzymes such as Alox12, Alox12e, and Alox15 reportedly have 12S-LOX activity in mice^36–38^. Alox5 was also reported to have weak 12S-LOX activity^39^. qRT-PCR analysis reliably detected Alox12 mRNA, but not the others, in the nucleus accumbens, although its levels were unaltered by chronic social defeat stress (Fig. 8a). To identify a cellular source of 12-LOX metabolites, we performed in situ hybridization and found Alox12 expression in blood vessel-like structures throughout the brain (Fig. 8b). Although its endothelial specificity remains to be histologically validated by the colocalization with an endothelial marker, the analyses of publicly available datasets (DropViz; http://dropviz.org/) of single cell RNA-seq of the mouse striatum, including the nucleus accumbens, showed Alox12 expression enriched in endothelial cells (Fig. 8c). Alox12 expression was also enriched in endothelial cells in other brain regions, including the frontal cortex and the hippocampus (Supplementary Fig. 1a,b). These findings suggest that the amounts of 12S-LOX metabolites derived from brain vasculature were increased in the nucleus accumbens of stress-resilient mice.

Given the notion that antidepressants may induce stress resilience, we examined whether the antidepressant imipramine would alter brain lipid metabolites similarly as chronic social defeat stress did in stress-resilient mice. We confirmed the antidepressant-like effects of imipramine on the immobility time in the forced swim test (Supplementary Fig. 2a). However, neither the forced swim test nor imipramine altered brain lipid metabolites, including 12-HETE and 12-HEPE, in the nucleus accumbens (Supplementary Fig. 2b).

## Discussion

In this study, we found that chronic social defeat stress increased the amounts of 12-LOX metabolites, 12-HETE and 12-HEPE, specifically in the nucleus accumbens in mice. Their increase was associated with stress resilience as stress-resilient mice showed the larger increase than stress-susceptible mice. Their increase occurred 1 week, but not immediately, after the stress, suggesting that it was not the direct effects of chronic social defeat stress. The chiral analysis demonstrated that the amount of the S isomer 12S-HETE was increased in the nucleus accumbens of stress-resilient mice, indicating the role of 12S-LOX activity. Among the enzymes having 12S-LOX activity, only Alox12 mRNA was reliably detected in the brain and enriched in the brain vasculature. These findings suggest that chronic social defeat stress induces a late increase in the amounts of 12S-LOX metabolites derived from the brain vasculature in a manner associated with stress resilience.

Previous studies have established the roles of the AA metabolite prostaglandin E_2_ and its receptor EP1 in chronic social defeat stress-induced behavioral changes^5,18,21,40^. Upon the stress, PGE_2_ is synthesized via cyclooxygenase (COX)-1 expressed in microglia and attenuates the dopaminergic pathway to the prefrontal cortex via EP1, leading to depressive behavior^5^. Notably, the amount of PGE_2_ increased immediately after the first and last stress exposure in chronic social defeat stress, whereas the present study showed no increase in the amount of PGE_2_ 1 week after the last stress exposure. This immediate PGE_2_ synthesis contrasts with the late synthesis of 12S-LOX metabolites, suggesting that the regulation of lipid mediator synthesis changes over 1 week after the stress. Since 12S-LOX metabolites were selectively upregulated among multiple lipid metabolites synthesized with different synthases, 12S-LOX activity could be selectively augmented. Since Alox12 expression was unaltered, its posttranscriptional modification could be involved. Among lipoxygenase family members, Alox5 is phosphorylated to be translocated to the nuclear membrane for its enzymatic function^41^. Alox12 phosphorylation was also reported although its role in regulating the enzymatic function remains to be elucidated^42,43^. Notably, 12-LOX metabolites increased in the nucleus accumbens, but not in the prefrontal cortex. Since Alox12 expression is similarly distributed in the two brain regions, the posttranscriptional modification of Alox12 could also be involved in this brain region specificity. In the prefrontal cortex, other lipid metabolites such as 15D-PGJ_2_, 18-HEPE, and 7-HDHA increased after chronic stress. The roles of the enzymes responsible for their synthesis in the behavioral effects of chronic stress warrant future investigation.

Given the endothelial expression of Alox12 in the brain, the 12S-LOX activity in the brain vasculature could increase along with the production of 12S-LOX metabolites. Chronic social defeat stress reportedly disrupts the barrier function of endothelial cells in the nucleus accumbens, facilitating the infiltration of proinflammatory cytokines along with depressive-like behaviors^14,15^. This barrier disruption was shown to be absent in stress-resilient mice. Endothelial cells in stress-resilient mice could have resilience-promoting machinery that augments the barrier function and activates 12S-LOX for long-term resilience. On the other hand, chronic stress-induced neuroinflammation could counteract such pro-resilience cascade in endothelial cells. Indeed, PGE_2_ derived from microglia was postulated to promote cerebrovascular injury via EP1 in endothelial cells^44^. Lipid mediator interactions in the brain vasculature warrant to be investigated for understanding the mechanism of stress resilience.

It should be noted that 12S-LOX metabolites increased 1 week, but not on the next day after chronic stress when stress resilience was assessed. This finding indicates that these metabolites are not necessary for stress resilience as measured by the level of social avoidance at that time point. However, these metabolites could exert its resilient effect on other behaviors induced by chronic stress as chronic stress induces not only social avoidance but also elevated anxiety and cognitive disturbances. Besides, the brain-wide pattern of neuronal activity reportedly changes over time after the cessation of chronic stress. Furthermore, it was reported that a subset of defeated mice showed social avoidance with a 2-week delay along with a different pattern of neuronal activity, compared with the mice that showed immediately-induced social avoidance. Thus, accumbal 12S-LOX metabolites could be involved in this delayed social avoidance. The mice lacking the enzymes with 12S-LOX activity, Alox12 and Alox15, would be useful for behavioral experiments to test these possibilities, although their double knockout mice need to be created newly as the two genes are apposed and genetically linked.

We previously reported that acute social defeat stress augments stress resilience through the prefrontal dopaminergic pathway. Thus, whether 12S-LOX metabolites are also upregulated under acute stress is an interesting question, although there is no method of defining stress resilience after acute stress as acute social defeat stress does not reliably induce social avoidance. We also examined the effect of acute treatment with the antidepressant imipramine, which did not affect the amounts of lipid metabolites in an hour. However, chronic antidepressant treatment is more relevant to the therapeutic effect in clinical settings and could have different effects on lipid metabolisms from its acute treatment.

Despite the upregulation of 12-HETE and 12-HEPE in stress-resilient mice, their causal roles for stress resilience remain unclear. Nonetheless, 12-LOX metabolites could attenuate chronic stress-induced neuroinflammation via multiple mechanisms. It was reported that 12-HEPE suppresses keratinocyte CXCL1/2 expression via retinoid X receptor α, inhibiting neutrophil recruitment and allergic skin inflammation^45^. This lipid metabolite also ameliorates atherosclerosis by blocking macrophage transformation to foam cells via peroxisome proliferator-activated receptor (PPAR) γ^46^. 12S-LOX is also involved in the synthesis of 14S-hydro(peroxy)-docosahexaenoic acid (14S-HpDHA), a precursor of maresin-1/2, from DHA^47^. Maresins are one class of anti-inflammatory, pro-resolving mediators and reportedly prevent inflammatory responses in various disease models in mice, including experimental autoimmune encephalomyelitis and inflammatory pain. Maresin-1 reportedly promotes phagocytosis and efferocytosis via its G protein-coupled receptor LGR6 in phagocytes^48^. 12S-LOX metabolites such as 12-HEPE and maresins could act on microglia, preventing inflammatory responses and promoting homeostatic functions for stress resilience. Whether 12-LOX metabolites have direct effects on neurons, other glial cells, and endothelial cells remains to be studied.

Resilience to mental illness was initially postulated by epidemiological observations that not all people who experienced lifetime trauma such as child maltreatment and war experience develop mental illness later in life^49^. However, biological mechanisms of resilience remain uncovered until recent studies on individual variability of susceptibility to chronic social defeat stress in mice^9,33^. These studies have identified pro-resilient mechanisms in the brain to prevent stress-induced depressive-like behavior. Among these mechanisms, the nucleus accumbens is one of key brain areas for stress resilience as stress resilience requires the activity of dopamine neurons projecting to the nucleus accumbens to be blocked by the upregulation of potassium channels^33^. Neuronal mechanisms for individual variability of stress susceptibility, such as BDNF-TrkB signaling and epigenetic regulation of neuronal morphology, have been elucidated^6,7,11,50^. However, given the presence of resilience-associated lipid metabolites from the brain vasculature suggested in this study, inflammation-related non-neuronal mechanisms of stress resilience as well as their impacts on neural circuits will be important for understanding brain mechanisms of stress resilience.

## Supplementary Information

Supplementary Figures.

## Data Availability

The datasets generated during and/or analyzed during the current study are available from the corresponding author on reasonable request.
