# Supplementary material for: Chronic social defeat stress increases the amounts of 12-lipoxygenase lipid metabolites in the nucleus accumbens of stress-resilient mice
Source: Sci Rep. 2022 Jul 5;12:11385. doi: 10.1038/s41598-022-15461-7 (PMC9256733; doi:10.1038/s41598-022-15461-7)
Supplement: Supplementary file 1 — Supplementary Figures. [file 41598_2022_15461_MOESM1_ESM.docx]

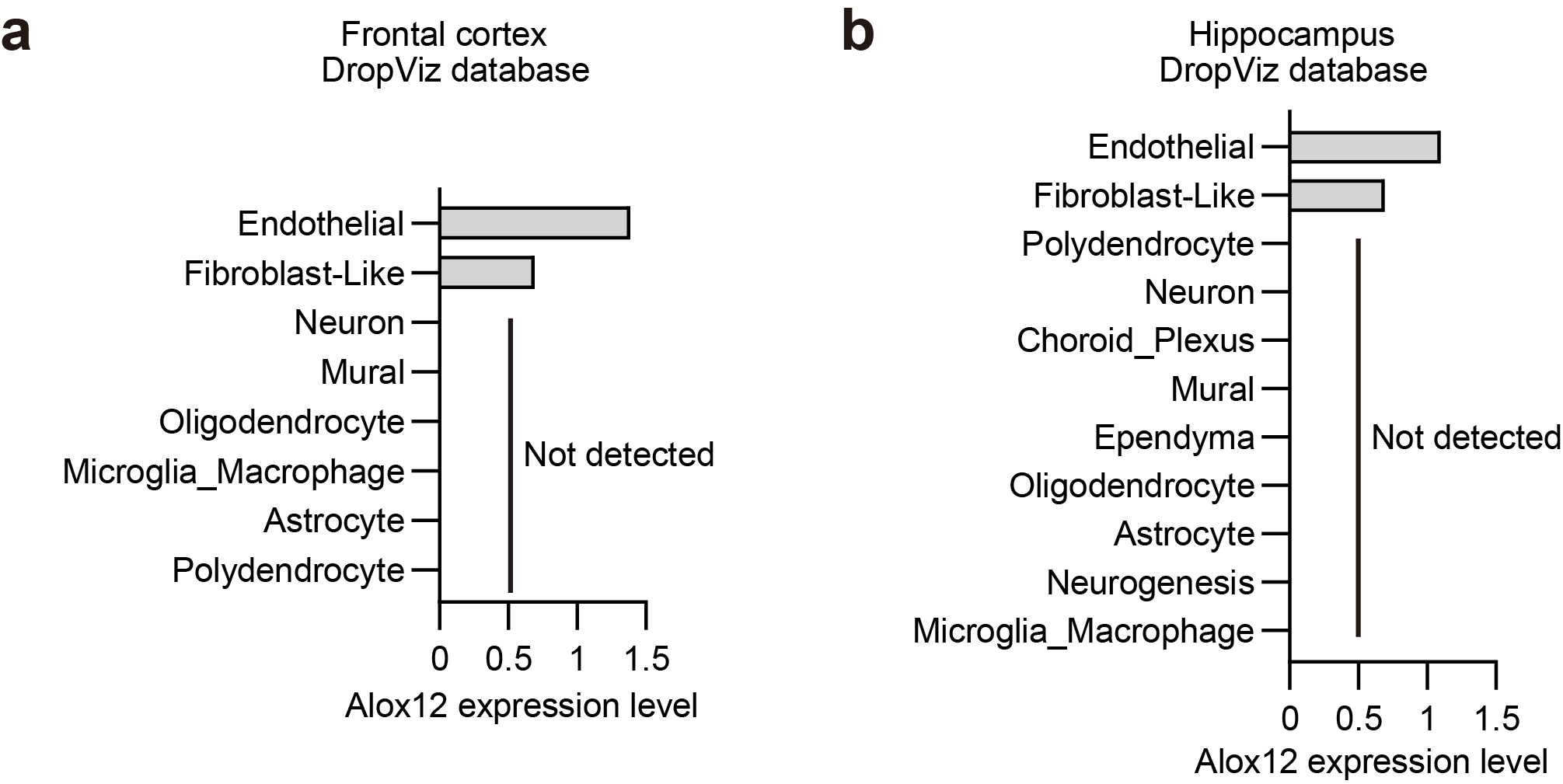


**Supplementary Figure 1. Alox12 expression was enriched in endothelial cells in the frontal cortex and the hippocampus.**

(a,b) Alox12 mRNA expression in endothelial cells in the frontal cortex (a) and in the hippocampus (b). Publicly available data of single cell RNA-seq were analyzed with the interactive online software DropViz.


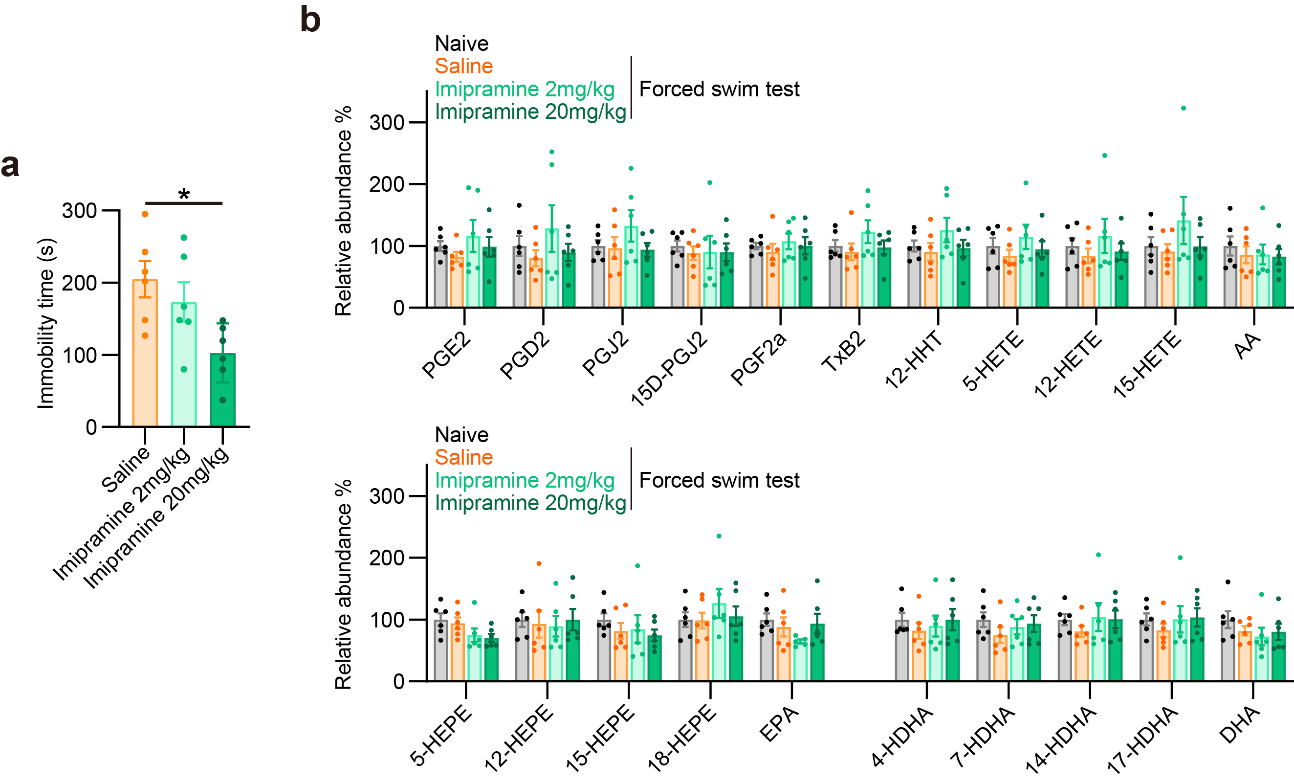


**Supplementary Figure 2. The antidepressant imipramine unaltered lipid metabolites in the nucleus accumbens.**

(a) Immobility time during the forced swim test. Mice were intraperitoneally injected either with saline as control or imipramine at 2 or 20 mg/kg 30 min before the forced swim test. **P*<0.05 for the comparison between Saline and Imipramine 20 mg/kg. ns, not significant. (b) The amounts of lipid metabolites in the nucleus accumbens of naïve mice that did not receive either the forced swim test or drug administration and stressed mice that received the forced swim test with administration of either saline or imipramine (2 or 20 mg/kg). The number of samples in each group is 6. No significant difference was found for Tukey’s multiple comparisons test.
